# An expanded cysteine‐rich receptor‐like kinase gene cluster functionally differentiates in drought, cold, heat, and pathogen stress responses in rice

**DOI:** 10.1111/pbi.14381

**Published:** 2024-05-19

**Authors:** Tiantian Ye, Huaijun Wang, Chengjing An, Haifu Tu, Lingqun Zhang, Dan Hu, Haiyan Xiong, Lizhong Xiong

**Affiliations:** ^1^ National Key Laboratory of Crop Genetic Improvement, Hubei Hongshan Laboratory Huazhong Agricultural University Wuhan China

**Keywords:** CRKs, gene cluster, CRISPR/Cas9, stress resistance, rice

Receptor‐like kinases (RLKs) are crucial for plant stress responses. Cysteine‐rich receptor‐like kinases (CRKs), a significant RLK subfamily, typically possess a signal peptide, one to four extracellular DUF26 domains, a transmembrane domain, and an intracellular serine/threonine kinase domain (Chen, 2001). *CRKs* are land plant‐specific, being present in vascular plants but absent in bryophytes and algae. They have expanded evolutionarily through tandem duplications (Vaattovaara *et al*., 2019). In Arabidopsis, *CRKs* exhibit responses to various environmental stimuli, influencing stress tolerance (Bourdais *et al*., 2015; Zeiner *et al*., 2023; Zhang *et al*., 2023). For example, *AtCRK2* enhances salt tolerance and immunity (Hunter *et al*., 2019; Kimura *et al*., 2020); *AtCRK5* and *AtCRK22* respond to *Verticillium dahliae* toxins (Zhao *et al*., 2022); *AtCRK4* contributes to drought tolerance (Zhao *et al*., 2023). Despite the known importance of *CRKs*, a comprehensive analysis of their functions in rice has been lacking. This study aimed to systematically investigate the phylogeny of the rice *CRK* family, and explore the functional divergence of an expanded *OsCRK* cluster in environmental adaptability.

Using AtCRK2 as a reference, we identified 54 CRK homologues in the rice, categorized into four phylogenetic groups (Figure 1a–c). Groups I and II align closely with AtCRK2, representing the basal clade with less divergence. Groups III and IV, forming the variable clade, show distinct phylogenetic clustering, indicating recent evolutionary divergence. Notably, chromosome 7 (Chr7) harbours 61.1% (33 out of 54) of *OsCRKs*, with 27 *OsCRKs* densely arrayed within the 20.96–21.44 Mb region, forming a significant *OsCRK* cluster (Figure S1). OsCRK33 and OsCRK36 within this cluster may be pseudo‐CRK*s*, due to lacking the extracellular DUF26 domain and transmembrane region (Figure 1b). Meanwhile, a small cluster on Chr12 exhibits a simpler gene structure (Figure S1), potentially representing ancestral *OsCRK*s (Figure 1c). These clusters likely formed through gene tandem duplication events, which played a major driving force for *CRK* evolution (Vaattovaara *et al*., 2019).

**Figure 1:**
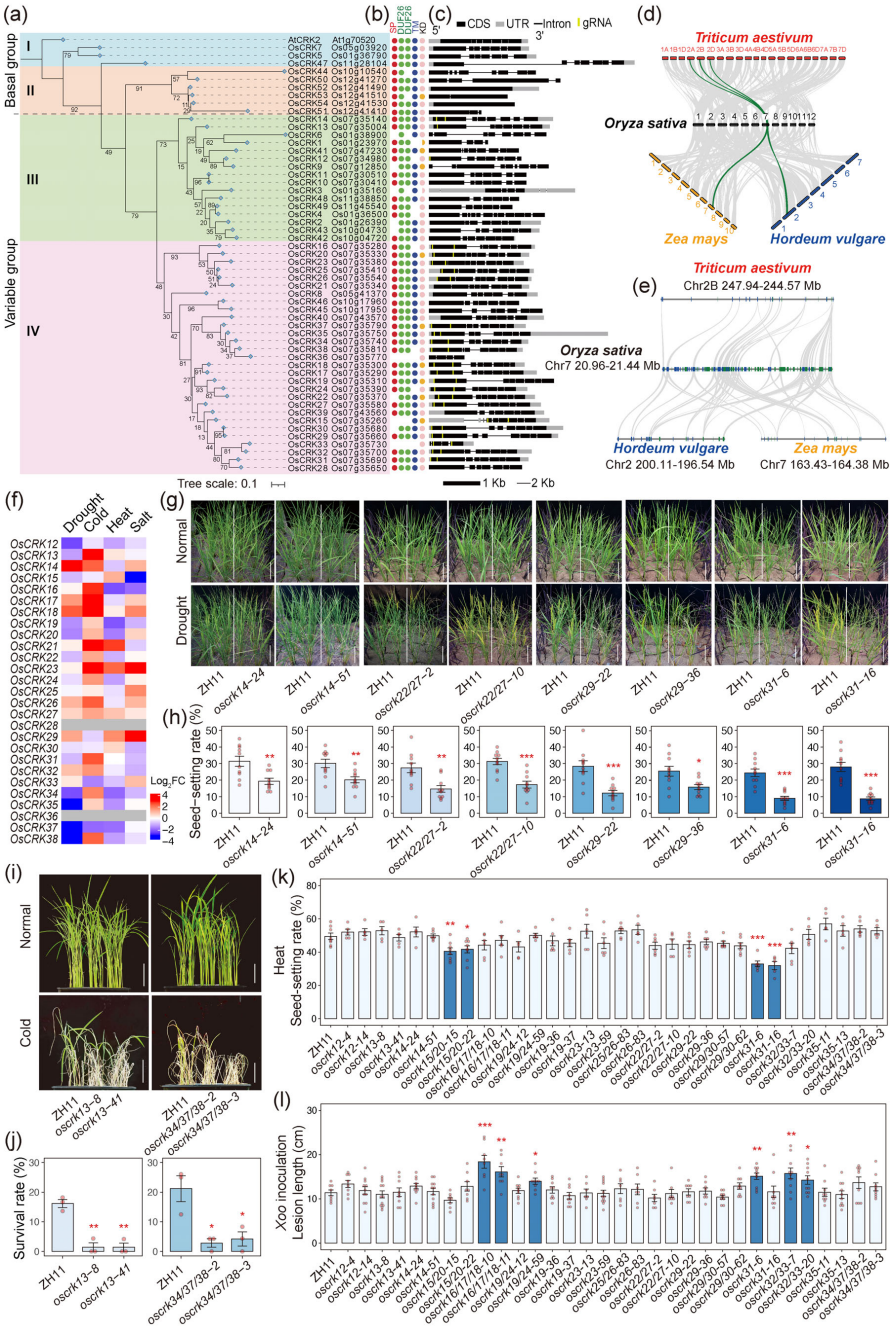
Genome‐wide identification and functional analysis of *OsCRKs* in rice stress tolerance. (a) Phylogenetic tree shows evolutionary relationships among 54 OsCRKs, categorized into four phylogenetic groups. Groups I and II belong to the basal clade, while groups III and IV belong to the variable clade. Bootstrap values (from 1000 replicates) are shown at branch nodes. Scale bar = 0.01 substitution per site. (b) Domain composition of OsCRKs: signal peptide (SP, red), domain of unknown function 26 (DUF26, green), transmembrane domain (TM, blue), kinase domain (KD, pink/orange). (c) Gene structures of *OsCRKs* and guide RNAs for gene editing. Coding sequences (CDS), untranslated regions (UTR), introns and guide RNAs (gRNA) are represented by black/grey strips and black/yellow lines. Scale bars: 1 kb (black strip), 2 kb (black line). (d, e) Collinearity analysis reveals similarities between rice and wheat (*Triticum aestivum*), maize (*Zea mays*), and barley (*Hordeum vulgare*) (d), particularly within the *OsCRK* cluster on chromosome 7 (e). (f) Gene expression profile of *OsCRKs* within the chromosome 7 cluster under drought, cold, heat, and salt treatments. (g, h) Field assessments show drought sensitivity (g) and lower seed‐setting rates (h) of *oscrk14*, *oscrk22/27*, *oscrk29*, and *oscrk31* compared with ZH11 during panicle development. Scale bars, 20 cm. Data = means ± s.e. (*n* = 10 plants). (i, j) Cold‐sensitivity (i) and survival rates (j) of *oscrk13* and *oscrk34/37/38* at the seedling stage. Scale bars, 3 cm. Data = means ± s.e. (*n* = 3 biological replicates). (k) Seed‐setting rates of *oscrks* and ZH11 under high‐temperature field conditions. Data = means ± s.e. (*n* ≥ 5 plants). (l) Lesion lengths of *oscrks* and ZH11 after inoculation with *Xanthomonas oryzae* pv. *oryzae* (*Xoo*) strain PXO99A. Data = means ± s.e. (*n* ≥ 7 leaves). Statistical significance is denoted by asterisks (*P < 0.05, **P < 0.01, ***P < 0.001).

To explore the evolutionary conservation of *CRK* gene clusters in crops, we conducted a collinearity analysis involving rice, barley, wheat, and maize (Figure 1d). The locus on rice Chr7 containing the *CRK* cluster has high collinearity with barley Chr2, wheat chromosome 2A/2B/2D, and maize Chr7 (Figure 1d), indicating the potential presence of *CRK* clusters before ancestral divergence. Notably, the rice collinear *CRK* cluster on Chr7 shows substantial expansion compared with those in barley, wheat, and maize (Figure 1e). This expansion raises questions about whether tandem expansion drives CRK functional diversification, such as subfunctionalization and neofunctionalization in rice.

Using CRISPR/Cas9, we generated large‐scale knockout mutants within the *OsCRK* cluster on Chr7. Guide RNAs targeting individual or multiple *OsCRKs* produced mutants for 25 *OsCRKs* (Figure 1c and Table S1). Comparative analysis of the morphological and developmental traits between mutants and the wild‐type (Zhonghua 11) during planting and harvesting stages revealed no significant differences (Figure S2), suggesting potential functional redundancy or indirect involvement of these *OsCRKs* in rice growth and development.

We further investigated the stress‐responsive expression patterns of *OsCRKs* within the cluster on Chr7 and found that numerous members exhibited responses to various abiotic stresses (Figure 1f). Drought stress screening showed increased drought sensitivity in mutants like *oscrk22/27*, *oscrk29*, *oscrk31*, and *oscrk35* at the seedling stage (Figure S3). During panicle development stage, *oscrk14*, *oscrk22/27*, *oscrk29*, and *oscrk31* mutants showed earlier wilting (Figure 1g) under drought stress and reduced seed‐setting rates after recovery (Figure 1h). Low temperature reduced survival rates in *oscrk13* single mutant and *oscrk34/37/38* triple mutant (Figure 1i,j). Natural high‐temperature conditions in the field led to lower seed‐setting rates in *oscrk15/20* and *oscrk31* mutants (Figure 1k and Figure S4). Upon *Xanthomonas oryzae* pv. *oryzae* (*Xoo*) strain inoculation, *oscrk16/17/18* triple mutant and *oscrk32/33* double mutant exhibited longer lesions compared with the wild‐type (Figure 1l), indicating roles in rice disease resistance. Overall, *OsCRK31* regulates multiple stress tolerances like drought and heat, while others like *OsCRK22/27* and *OsCRK29* specialize in drought resistance, *OsCRK13* and *OsCRK34/37/38* in low‐temperature tolerance, *OsCRK15/20* in high‐temperature tolerance, and *OsCRK16/17/18* and *OsCRK32/33* in bacterial disease resistance.

In summary, our study systematically analysed the rice CRK family, revealing that *OsCRK* clusters formed through tandem duplication, a process conserved in grasses but expanded in rice. *OsCRKs* within the cluster on Chr7 exhibit diverse roles in responding to drought, cold, heat, and pathogen stresses. The tandem amplification potentially contributed to the diverse evolution of *OsCRK* functions, improving rice's environmental adaptability. This research provides novel insights into the evolution and function of the *OsCRKs*, offering potential genetic resources for rice stress resistance improvement. These findings also have broader implications for stress resistance research in other crops.

## Conflict of interest

The authors declare no conflict of interest.

## Author contributions

L.X. and H.X. designed the project. T.Y. generated the mutants. H.W. analysed the data. T.Y., H.W., C.A., H.T., and L.Z. performed the experiments. T.Y., H.W., H.X., and L.X. wrote the manuscript.

## Supporting information


**Figure S1** Chromosomal distribution of *OsCRK* genes.
**Figure S2** Effect of *oscrk* mutations on plant height and tiller numbers.
**Figure S3** Effects of *oscrk* mutations on drought tolerance at the seedling stage.
**Figure S4** Temperature trends during the rice reproductive stage under natural heat stress conditions in the field.

**Table S1** Guide RNAs and gene editing results of *oscrk* mutants.

**Table S2** List of PCR primers for genotyping of *oscrk* mutants.

## Data Availability

The data that support the findings of this study are openly available in Rice cold treatment RNA‐seq at https://www.ncbi.nlm.nih.gov/, reference number PRJNA1102144.

## References

[pbi14381-bib-0001] Bourdais, G. , Burdiak, P. , Gauthier, A. , Nitsch, L. , Salojärvi, J. , Rayapuram, C. , Idänheimo, N. *et al*. (2015) Large‐scale phenomics identifies primary and fine‐tuning roles for CRKs in responses related to oxidative stress. PLoS Genet. 11, e1005373. 26197346 10.1371/journal.pgen.1005373 PMC4511522

[pbi14381-bib-0002] Chen, Z. (2001) A superfamily of proteins with novel cysteine‐rich repeats. Plant Physiol. 126, 473–476. 11402176 10.1104/pp.126.2.473 PMC1540112

[pbi14381-bib-0003] Hunter, K. , Kimura, S. , Rokka, A. , Tran, H.C. , Toyota, M. , Kukkonen, J.P. and Wrzaczek, M. (2019) CRK2 enhances salt tolerance by regulating callose deposition in connection with PLDα1. Plant Physiol. 180, 2004–2021. 31118265 10.1104/pp.19.00560 PMC6670071

[pbi14381-bib-0004] Kimura, S. , Hunter, K. , Vaahtera, L. , Tran, H.C. , Citterico, M. , Vaattovaara, A. , Rokka, A. *et al*. (2020) CRK2 and C‐terminal phosphorylation of NADPH oxidase RBOHD regulate reactive oxygen species production in Arabidopsis. Plant Cell, 32, 1063–1080. 32034035 10.1105/tpc.19.00525 PMC7145479

[pbi14381-bib-0005] Vaattovaara, A. , Brandt, B. , Rajaraman, S. , Safronov, O. , Veidenberg, A. , Luklová, M. , Kangasjärvi, J. *et al*. (2019) Mechanistic insights into the evolution of DUF26‐containing proteins in land plants. Commun. Biol. 2, 56. 30775457 10.1038/s42003-019-0306-9 PMC6368629

[pbi14381-bib-0006] Zeiner, A. , Colina, F.J. , Citterico, M. and Wrzaczek, M. (2023) CYSTEINE‐RICH RECEPTOR‐LIKE PROTEIN KINASES: their evolution, structure, and roles in stress response and development. J. Exp. Bot. 74, 4910–4927. 37345909 10.1093/jxb/erad236

[pbi14381-bib-0007] Zhang, Y. , Tian, H. , Chen, D. , Zhang, H. , Sun, M. , Chen, S. , Qin, Z. *et al*. (2023) Cysteine‐rich receptor‐like protein kinases: emerging regulators of plant stress responses. Trends Plant Sci. 28, 776–794. 37105805 10.1016/j.tplants.2023.03.028

[pbi14381-bib-0008] Zhao, J. , Sun, Y. , Li, X. and Li, Y. (2022) CYSTEINE‐RICH RECEPTOR‐LIKE KINASE5 (CRK5) and CRK22 regulate the response to *Verticillium dahliae* toxins. Plant Physiol. 190, 714–731. 35674361 10.1093/plphys/kiac277 PMC9434262

[pbi14381-bib-0009] Zhao, M. , Li, M. , Huang, M. , Liang, C. , Chen, D. , Hwang, I. , Zhang, W. *et al*. (2023) The cysteine‐rich receptor‐like kinase CRK4 contributes to the different drought stress response between Columbia and Landsberg *erecta* . Plant Cell Environ. 46, 3258–3272. 37427814 10.1111/pce.14665

