# Supplementary material for: An expanded cysteine‐rich receptor‐like kinase gene cluster functionally differentiates in drought, cold, heat, and pathogen stress responses in rice
Source: Plant Biotechnol J. 2024 May 19;22(10):2672–4. doi: 10.1111/pbi.14381 (PMC11536441; doi:10.1111/pbi.14381)
Supplement: Supplementary file 1 — Figure S1 Chromosomal distribution of OsCRK genes. Figure S2 Effect of oscrk mutations on plant height and tiller numbers. Figure S3 Effects of oscrk mutations on drought tolerance at the seedling stage. Figure S4 Temperature trends during the rice reproductive stage under natural heat stress conditions in the field. [file PBI-22-2672-s003.docx]

**Supplemental methods**

**Plant materials and growth conditions**

The *oscrk* mutants were generated using the CRISPR/Cas9 genome editing system. Initially, the guide RNA (gRNA) sequences targeting the *OsCRK* genes were identified through the CRISPR-PLANT website (http://omap.org/crispr/). DNA fragments containing the gRNA sequences were amplified through overlap extension PCR and subsequently cloned into the CRISPR vector pRGEB32 (Xie et al., 2015) or TKC (He et al., 2018), with each construct containing one to four gRNAs. The constructed vectors were transformed into *Agrobacterium tumefaciens* strain EHA105, which was then used to mediate the transformation of the ZH11 cultivar (*Oryza sativa* ssp. *japonica*). The Cas9-free and homozygous T_2_ generation were used for further experiments. Guide RNAs used and gene editing results of *oscrk* mutants in this study are listed in Table S1. The primers for genotyping of *oscrk* mutants are listed in Table S2. All the rice plants were cultivated in the experimental fields of Huazhong Agricultural University (Wuhan, Hubei, China).

**Phylogenetic Analysis**

The protein sequences of Arabidopsis AtCRK2 were used as a reference to identify homologous CRK sequences in *Oryza sativa* via BLASTP search against the protein sequence from Rice Genome Annotation Project database (http://rice.uga.edu/). The retrieved sequences were then verified for the presence of conserved domains characteristic of CRK proteins using the SMART databases (<http://smart.embl-heidelberg.de/>). Multiple sequence alignment of the CRK sequences was performed using Muscle software. Phylogenetic trees were constructed from the aligned sequences using the Molecular Evolutionary Genetics Analysis (MEGA, version 7.0) software, with the Maximum-likelihood method, and with 1000 bootstrap replicates to assess the reliability of the inferred phylogenetic relationship.

**Chromosome Location and Collinearity Analysis**

To elucidate the genomic distribution of *CRK* genes within the rice genome, we determined and visualized the physical locations of the *CRK* genes on the rice chromosomes using MapChart software. To detect evolutionary relationships and potential gene duplication among rice, maize, wheat, and barley, we performed a collinearity analysis across *Oryza sativa*, *Triticum aestivum*, *Zea mays*, and *Hordeum vulgare* using the MCScanX software.

**Gene Expression Analysis**

To investigate the gene expression profiles of rice under various stress conditions, we collected the RNA-Seq data from multiple sources. For drought, salt, and heat stress, we accessed datasets from the NCBI Sequence Read Archive with the accession numbers PRJNA527848, PRJNA546269, and PRJNA810084, respectively. For cold stress, we conducted RNA-Seq experiment. Total RNA was isolated from rice seedlings at the four-leaf stage that had been exposed to cold conditions (4℃ for 2 days) using *TransZol* RNA Kit (TransGen Biotech). Library construction and sequencing were performed by Novogene Inc. (Tianjin, China) with HiSeq-PE150 (Illumina Inc., San Diego, CA, USA) using the paired-end sequencing strategy. The raw reads sequencing reads obtained were subjected to a stringent quality control process using fastp software to ensure that only high-quality, clean reads were retained for further analysis. These clean reads were then aligned to the reference rice genome (Nipponbare, MSU 7.0) using HISAT2 (version 2.2.1). The aligned reads were quantified at the gene level using featureCounts (version 1.5.0). The differential expression analysis was performed using the DESeq2 R package (version 1.30.1). The raw sequencing files of the cold treatment have been deposited in the National Center for Biotechnology Information (PRJNA1102144). The log_2_ fold change (log_2_FC) values of the *OsCRKs* under the drought, salt, heat, and cold stress conditions compared to normal conditions were used for heatmap analysis. The heatmap was performed by using the Complex Heatmap package (version 2.18.0) in R (version 4.3.2).

**Drought stress treatment**

For drought stress treatment at the seedling stage, the seeds of the *oscrk* mutants and the wild type (ZH11) were germinated on half-strength Murashige and Skoog medium for five days. Uniformly germinated seeds were transferred to barrels, with each barrel containing 12 mutant plants and 12 ZH11 plants as controls. Drought treatments were conducted at the four-leaf stage by withholding water for 7-10 days. Once a significant difference in wilting was observed between the mutants and ZH11, re-watering was conducted for 7-10 days, after which survival rates were calculated. Plants exhibiting green leaves and regenerating shoots were considered surviving plants.

For drought stress treatment at the panicle development stage, the *oscrk* mutants and the wild type (ZH11) were initially sown in seedling nurseries. Twenty-five-day-old seedlings were transplanted to the field. Each experimental plot consisted of two sections: the left side with the wild-type ZH11 and the right side with mutant plants. Five plants of each genotype were planted per row, with two rows for each family, resulting in ten replicates. Irrigation was ceased at the end of the tillering stage. A movable rain shelter was utilized on rainy days to shield the experimental plots from rainwater. Phenotypic differences were documented by capturing photos as they became evident. When irreversible leaf rolling occurred, the drought stress was maintained for an additional 5 days. After this period, the drought stress was terminated, and the plants entered the recovery phase with re-watering until harvest. Seed-setting rates were recorded for evaluation.

**Cold stress treatment**

Uniformly germinated seeds of *oscrk* mutants and the wild type (ZH11) were transferred into bottomless 96-well plates placed within boxes. Fourteen-days old seedlings were subjected to cold stress at 4℃ for five days followed by recovery at 25℃ for one week. After the recovery period, the survival rates were evaluated. The plants with green leaves and regenerating shoots were considered as survived plants.

**Heat stress treatment**

The *oscrk* mutants and wild type (ZH11) were initially sown in seedling nurseries on May 5, 2021. After 25 days post-sowing, seedlings were transplanted into the field at Huazhong Agricultural University, Wuhan, China. Both the *oscrk* mutants and ZH11 began heading on July 20^th^, 2021. Throughout the reproductive phase, the plants experienced natural high-temperature stress. We collected the daily highest and lowest temperatures in Wuhan from 10 days before heading to 30 days after heading (July 1 to August 20, 2021), covering crucial reproductive stages such as panicle development, heading, flowering, and grain-filling (Figure S4). Upon reaching maturity, seed-setting rates were recorded for evaluation. Temperature data were obtained from the National Meteorological Information Center (https://data.cma.cn/data/).

**Pathogen inoculation**

The *oscrk* mutants and the wild type (ZH11) plants were grown in the field. The *Xanthomonas oryzae* pv. *oryzae* (*Xoo*) strain PXO99A was grown at 28 °C on a nutrient agar medium. At the panicle development stage, rice leaves were inoculated with PXO99A strain with OD_600nm_ = 0.5 by the leaf-clipping method (Hui et al., 2019). Disease severity was scored by measuring the lesion length on the 14^th^ day after inoculation.

**Statistical analysis**

All statistical analyses were conducted in R (V.4.3.1). Student’s two-tailed *t*-test was performed to compare differences in phenotype between the *oscrk* mutants and the wild type (ZH11).

**Supplemental figures**


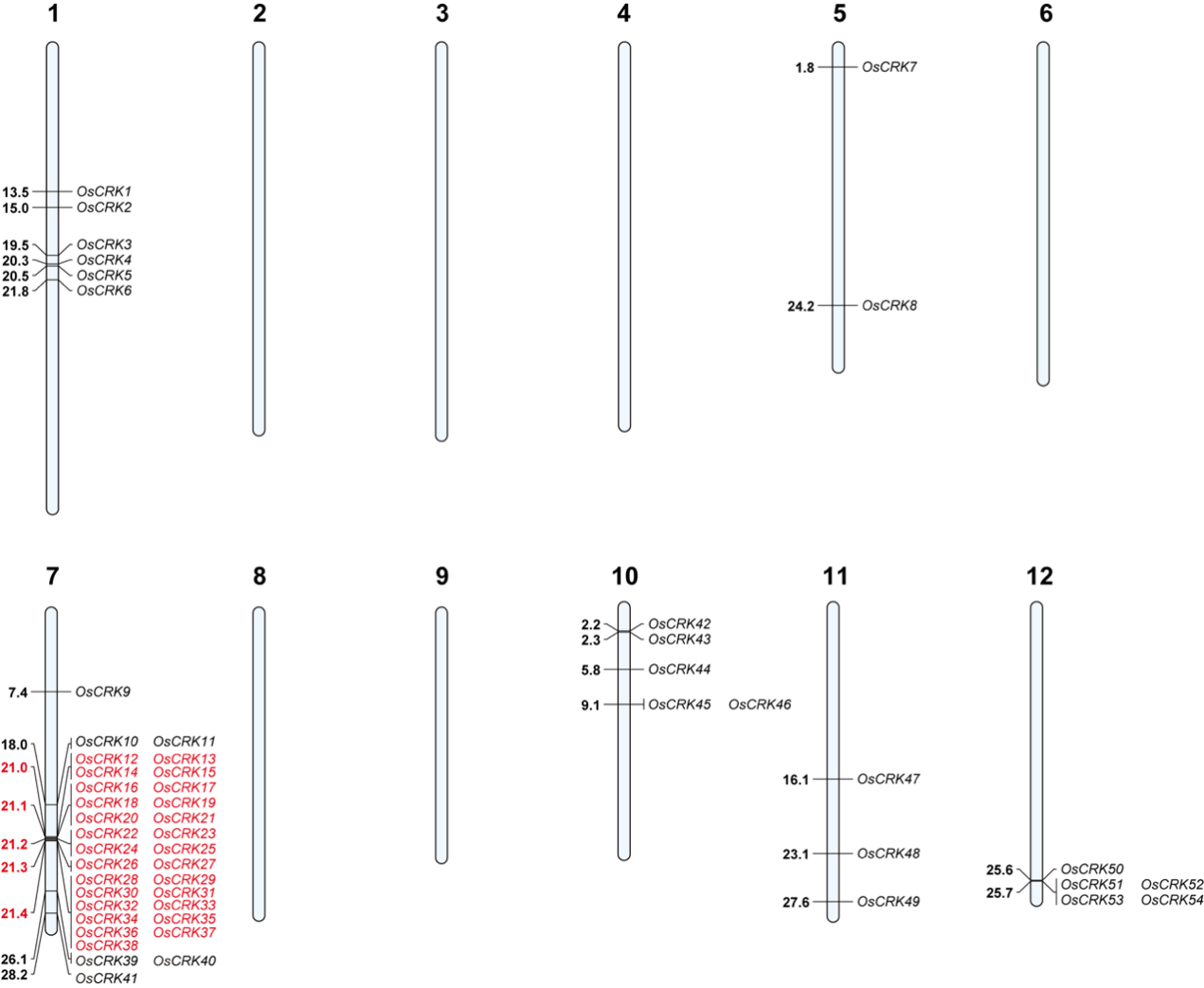


**Figure S1.** Chromosomal distribution of *OsCRK* genes. *OsCRKs* are distributed across chromosomes 1, 5, 7, 10, 11, and 12. The members of the *OsCRK* cluster on chromosome 7 (*OsCRK12-38*) are highlighted in red.


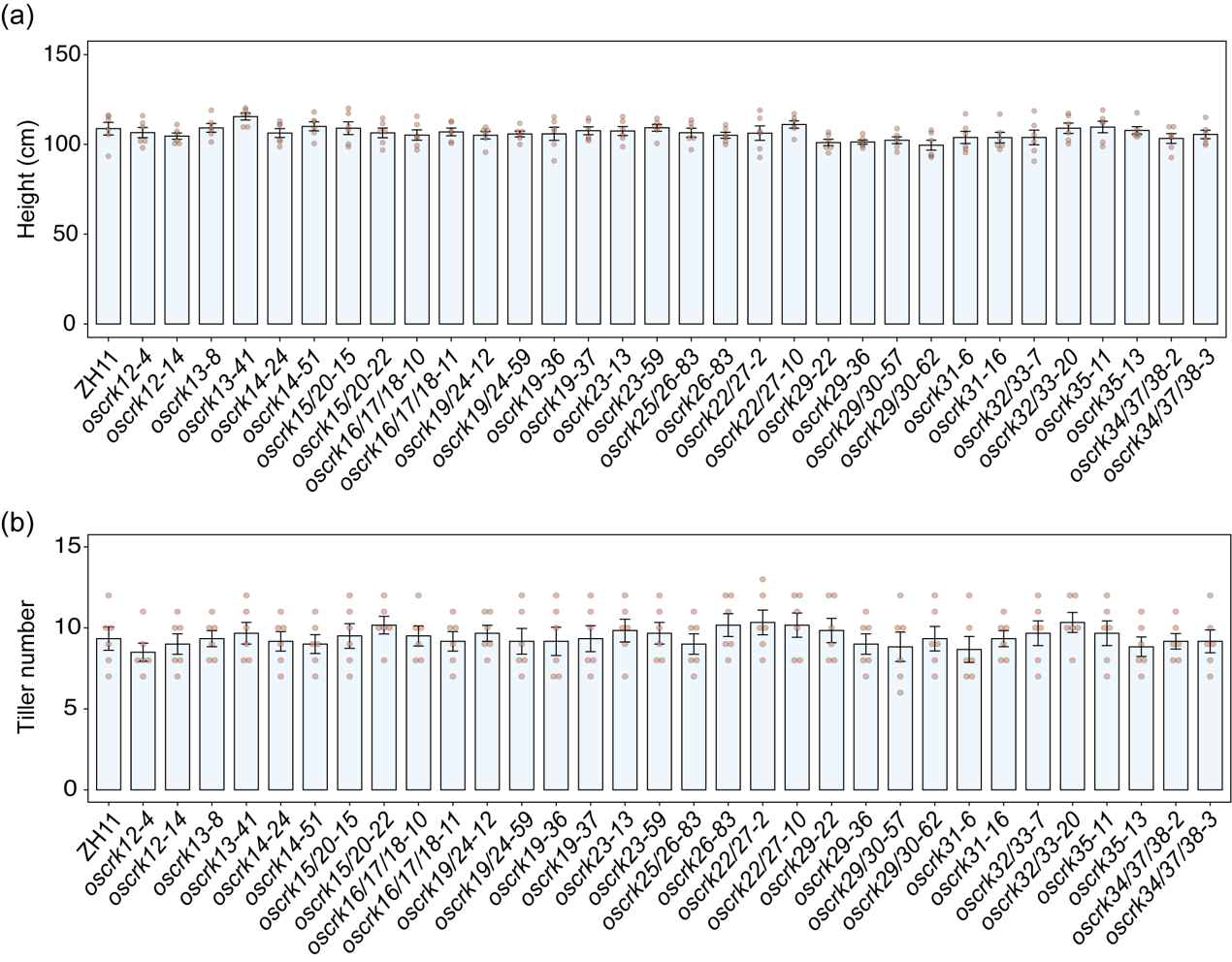


**Figure S2.** Effect of *oscrk* mutations on plant height and tiller numbers. (a) Comparison of plant height between *oscrk* mutants and ZH11 at the heading stage under normal growth conditions in the field. (b) Comparison of tiller numbers at the heading stage between *oscrk* mutants and ZH11 under normal growth conditions in the field. All plants were grown in experimental fields at Huazhong Agricultural University (Wuhan, Hubei, China). Data represent means ± s.e. (n = 6 plants).


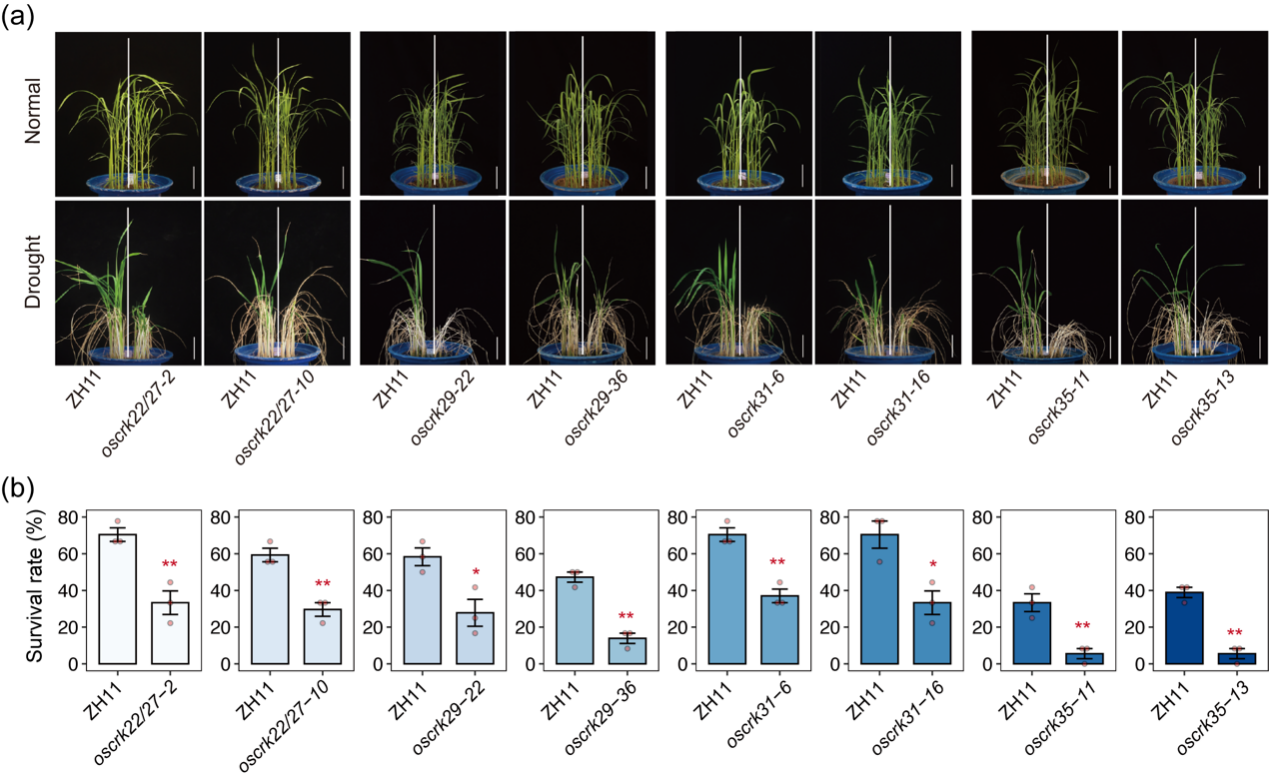


**Figure S3**. Effects of *oscrk* mutations on drought tolerance at the seedling stage. (a) Drought-sensitive phenotypes of *oscrk22/27*, *oscrk29*, *oscrk31,* and *oscrk35* compared to ZH11. Seedlings of the *oscrk* mutants were grown in barrels, each with the wild type (ZH11) plants as a control. At the four-leaf stage, drought stress treatment was conducted by removing the water supply for one week followed by recovery for one week. In each barrel, the left is the wild-type ZH11 and the right is the *oscrk* mutants. Phenotypes were recorded under normal conditions (up) and after recovery for one week (down). Scale bars, 5 cm. (b) Survival rates of *oscrk* mutants and ZH11 after one week of recovery. Plants with green leaves and regenerating shoots were considered as survived plants. Data represent means ± s.e. (n = 3 biological replicates). Asterisks indicate statistical significance by two-tailed Student's *t*-tests (**P* < 0.05, ***P* < 0.01).

**
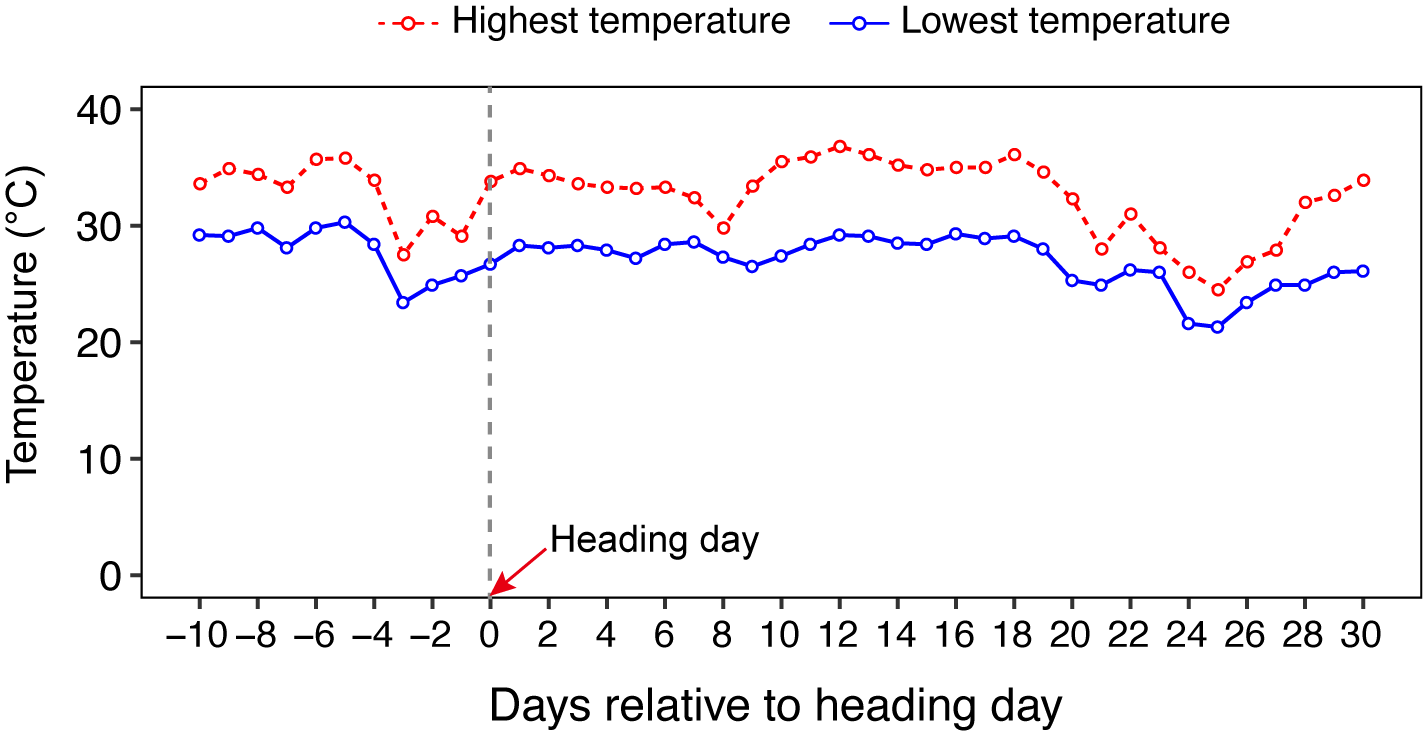
**

**Figure S4**. Temperature trends during the rice reproductive stage under natural heat stress conditions in the field. The heading date for *oscrk* mutants and ZH11 in Wuhan is July 20, 2021, indicated as Day 0 (marked with a dashed line and arrow). The daily highest and lowest temperatures were recorded from 10 days before heading to 30 days after heading, covering key reproductive stages including panicle development, heading, flowering, and grain filling.

**References**

He, Y., Zhu, M., Wang, L., Wu, J., Wang, Q., Wang, R., & Zhao, Y. (2018). Programmed Self-Elimination of the CRISPR/Cas9 Construct Greatly Accelerates the Isolation of Edited and Transgene-Free Rice Plants. *Mol Plant, 11*(9), 1210-1213. doi:10.1016/j.molp.2018.05.005

Hui, S., Liu, H., Zhang, M., Chen, D., Li, Q., Tian, J., . . . Yuan, M. (2019). The host basal transcription factor IIA subunits coordinate for facilitating infection of TALEs-carrying bacterial pathogens in rice. *Plant Sci, 284*, 48-56. doi:10.1016/j.plantsci.2019.04.004

Xie, K., Minkenberg, B., & Yang, Y. (2015). Boosting CRISPR/Cas9 multiplex editing capability with the endogenous tRNA-processing system. *Proc Natl Acad Sci U S A, 112*(11), 3570-3575. doi:10.1073/pnas.1420294112
