# Supplementary material for: An expanded cysteine‐rich receptor‐like kinase gene cluster functionally differentiates in drought, cold, heat, and pathogen stress responses in rice
Source: Plant Biotechnol J. 2024 May 19;22(10):2672–4. doi: 10.1111/pbi.14381 (PMC11536441; doi:10.1111/pbi.14381)
Supplement: Supplementary file 2 — Table S1 Guide RNAs and gene editing results of oscrk mutants. [file PBI-22-2672-s001.docx]

**Table S1. Guide RNAs and gene editing results of *oscrk* mutants.**

| ***oscrk* mutants** | **Lines** | **Guide RNAs used and gene editing results** | |
| --- | --- | --- | --- |
| ***oscrk12*** |  | *OsCRK12*-gRNA1 |  |
|  |  | ZH11: TTCCCTCCTAATGCTTCTCCTCTCTACCCC |  |
|  | *oscrk12-4* | *crk12*: TTCCCTCC--------GCTTCTCCTCTCTACCCC （-4 bp） |  |
|  | *oscrk12-14* | *crk12*: TTCCCTC----------GCTTCTCCTCTCTACCCC （-5 bp） |  |
| ***oscrk13*** |  | *OsCRK13*-gRNA1 |  |
|  |  | ZH11: ACCCGATCTCCTCCTTCTGTAACACGACGG |  |
|  | *oscrk13-8* | *crk13*: ACCCGA----------TCCTTCTGTAACACGACGG （-5 bp） |  |
|  | *oscrk13-41* | *crk13*: ACCCGA----TCCTCCTTCTGTAACACGACGG （-2 bp） |  |
| ***oscrk14*** |  | *OsCRK14*-gRNA1 | *OsCRK14*-gRNA2 |
|  |  | ZH11: CACCCGGGGCAACTTCACGGAGGGGAGCGC | ZH11: CTCCGCGCGCTCTGCGGCCGGGACCGCGAC |
|  | *oscrk14-24* | *crk14*: CACCCGGG-------------------------------------------------------------------------------------------------------CTCTGCGGCCGGGACCGCGAC （-224 bp） | |
|  | *oscrk14-51* | *crk14*: CACCCGGG**T**GCAACTTCACGGAGGGGAGCGC （+1 bp） | *crk14*: CTCCGCGC**T**GCTCTGCGGCCGGGACCGCGAC （+1 bp） |
| ***oscrk15/20*** |  | *OsCRK15*-gRNA1 | *OsCRK15*-gRNA2 |
|  |  | ZH11: AGCCATTGCATTGCCTATAGTCGCTGCAAT | ZH11: TCCTGATAGAAGCAATGTGCTGGATTGGTG |
|  | *oscrk15/20-15* | *crk15*: WT | *crk15*: TCCTGATAGAAGCAATGTGCTG--ATTGGTG （-1 bp） |
|  | *oscrk15/20-22* | *crk15*: WT | *crk15*: TCCTGATAGAAGCAATGTGCTG--ATTGGTG （-1 bp） |
|  |  | *OsCRK20*-gRNA1 | *OsCRK20*-gRNA2 |
|  |  | ZH11: CGCCGCGGCGCAGCAGACCATGCAGTTCTC | ZH11: CACCAAGAAGTACTTCGCCACGGCGGTGGA |
|  | *oscrk15/20-15* | *crk20*: CGCCGCGG--GCAGCAGACCATGCAGTTCTC （-1 bp） | *crk20*: CACCAAGA--GTACTTCGCCACGGCGGTGGA （-1 bp） |
|  | *oscrk15/20-22* | *crk20*: CGCCGCGG--GCAGCAGACCATGCAGTTCTC （-1 bp） | *crk20*: CAACA--------GTACTTCGCCACGGCGGTGGA （-10 bp） |
| ***oscrk16/17/18*** |  | *OsCRK16*-gRNA1 | *OsCRK16*-gRNA2 |
|  |  | ZH11: TCGCCTTCCTCGCCGTCGCGCTACCCGGTG | ZH11: GTGCAGTGCACGCCGGACATGTCGCCGGCC |
|  | *oscrk16/17/18-10* | *crk16*: TCGCC--------------------------------------CCGGTG （-19 bp） | *crk16*: WT |
|  | *oscrk16/17/18-11* | *crk16*: TCGCCTTCCTCGCCGTCGCGC**T**TACCCGGTG （+1 bp） | *crk16*: WT |
|  |  | *OsCRK17*-gRNA1 | *OsCRK17*-gRNA2 |
|  |  | ZH11: GCCCTCTTCGCGGCCGGTAGCCTCGGCACC | ZH11: TCCCAGTGCACGCCGGACATGTCGCCGGAC |
|  | *oscrk16/17/18-10* | *crk17*: WT | *crk17*: TCCCAGTGCACGCCGGACATGT**T**CGCCGGAC （+1 bp） |
|  | *oscrk16/17/18-11* | *crk17*: WT | *crk17*: TCCCAGTGCACGCCGGACATGT**A**CGCCGGAC （+1 bp） |
|  |  | *OsCRK18*-gRNA1 | *OsCRK18*-gRNA2 |
|  |  | ZH11: CGGCACCTACCAGTCCAACCTCGCCGGGCT | ZH11: TCCCAGTGCACGCCGGACATGTCGCCGGAC |
|  | *oscrk16/17/18-10* | *crk18*: CGGCACCTACCAGTCCAACCTC**A**GCCGGGCT （+1 bp） | *crk18*: WT |
|  | *oscrk16/17/18-11* | *crk18*: WT | *crk18*: TCCCAGTGCACGCCGGACATGT**T**CGCCGGAC （+1 bp） |
| ***oscrk19/24*** |  | *OsCRK19*-gRNA1 | *OsCRK19*-gRNA2 |
|  |  | ZH11: GTCCATGGCGCATCGCGCTGGCTGCCTCTC | ZH11: GTCGGAGTTCCCCAAGGTGTACAGCTGGGC |
|  | *oscrk19/24-12* | *crk19*: GTCCAT**G**GGCGCATCGCGCTGGCTGCCTCTC （+1 bp） | *crk19*: GTCGGAGTTCCCCAAGGTGTA--AGCTGGGC （-1 bp） |
|  | *oscrk19/24-59* | *crk19*: GTCCATGG--GCATCGCGCTGGCTGCCTCTC （-1 bp） | *crk19*: GTCGGAGTTCCCCAAGGT--------AGCTGGGC （-4 bp） |
|  |  | *OsCRK24*-gRNA1 | *OsCRK24*-gRNA2 |
|  |  | ZH11: TGCAACGACACGGCCGGCGAATTCCCGGCG | ZH11: CGCGGAGTTCCCCAAGGTGTACAGCTGGGC |
|  | *oscrk19/24-12* | *crk24*: WT | *crk24*: GTCGGAGTTCCCCAAGGTG-----------CTGGGC （-5 bp） |
|  | *oscrk19/24-59* | *crk24*: TGCAACGACACGGCCGGCGAAT**A**TCCCGGCG （+1 bp） | *crk24*: WT |
| ***oscrk19*** |  | *OsCRK19*-gRNA1 |  |
|  |  | ZH11: GTCCATGGCGCATCGCGCTGGCTGCCTCTC |  |
|  | *oscrk19-36* | *crk19*: GTCCATGG--GCATCGCGCTGGCTGCCTCTC （-1 bp） |  |
|  | *oscrk19-37* | *crk19*: GTCCATGG--GCATCGCGCTGGCTGCCTCTC （-1 bp） |  |
| ***oscrk23*** |  | *OsCRK23*-gRNA1 | *OsCRK23*-gRNA2 |
|  |  | ZH11: GTCCTCTTGCCGCCGTCGGCCACGCCGGCT | ZH11: TATAGGTTCATGGATTTCCTCAGTCCGGAT |
|  | *oscrk23-13* | *crk23*: WT | *crk23*: TATAGGTTCATGGATTTC--------GTCCGGAT （-4 bp） |
|  | *oscrk23-59* | *crk23*: GTCCTCTTGCCG------TCGGCCACGCCGGCT （-3 bp） | *crk23*: TATAGGTTCATGGATTTCCTC**A**AGTCCGGAT （+1 bp） |
| ***oscrk25/26*** |  | *OsCRK25*-gRNA1 | *OsCRK25*-gRNA2 |
|  |  | ZH11: GTCCTCACGCTGCCCTCGGCCGTGCGTGCC | ZH11: CTCCGGGCCCGGGGACGGCTGCGTGCTCCG |
|  | *oscrk25/26-83* | *crk25*: GTCCTCACG----GCCCTCGGCCGTGCGTGCC （-2 bp） | *crk25*: CTCCGGGC----GGGGACGGCTGCGTGCTCCG （-2 bp） |
|  |  | *OsCRK26*-gRNA1 | *OsCRK26*-gRNA2 |
|  |  | ZH11: CGATGCTCGCCGGCTATGTCTACGGTGGTG | ZH11: TCGACGCGAAGGGCGTCTGCCCCGGCGGCA |
|  | *oscrk25/26-83* | *crk26*: CGATGCTCGCCG-------------------------------------------------------------------------------------------------------------------------TGGTG （-491 bp） | |
| ***oscrk26*** |  | *OsCRK26*-gRNA1 | *OsCRK26*-gRNA2 |
|  |  | ZH11: CGATGCTCGCCGGCTATGTCTACGGTGGTG | ZH11: TCGACGCGAAGGGCGTCTGCCCCGGCGGCA |
|  | *oscrk26-83* | *crk26*: CGATGCTCGCCG-------------------------------------------------------------------------------------------------------------------------TGGTG （-491 bp） | |
| ***oscrk22/27*** |  | *OsCRK22*-gRNA1 | *OsCRK22*-gRNA2 |
|  |  | ZH11: CGCCGGAAATACCCCGATGGATCGCCGTCT | ZH11: CGCCACCTACTACGACCGGTGCATGGTCAG |
|  | *oscrk22/27-2* | *crk22*: CGCCGG--AATACCCCGATGGATCGCCGTCT （-1 bp） | *crk22*: CGCCACCT-------------ACCGGTGCATGGTCAG （-6 bp） |
|  | *oscrk22/27-10* | *crk22*: CGCCGG--AATACCCCGATGGATCGCCGTCT （-1 bp） | *crk22*: WT |
|  |  | *OsCRK27*-gRNA1 | *OsCRK27*-gRNA2 |
|  |  | ZH11: GCGCTGCTTCTCCTCGCGGCGCTCCCGGCG | ZH11: GCCGACTGCGTCGGCACCATCTTCCAGGAC |
|  | *oscrk22/27-2* | *crk27*: WT | *crk27*: GCCGACTGCGTCGGCACCATC**T**TTCCAGGAC （+1 bp） |
|  | *oscrk22/27-10* | *crk27*: WT | *crk27*: GCCGACTGCGTCGGCACCATC**T**TTCCAGGAC （+1 bp） |
| ***oscrk29*** |  | *OsCRK29*-gRNA1 |  |
|  |  | ZH11: GGCCATGCGACGCCGACGAAATCTCACTCC |  |
|  | *oscrk29-22* | *crk29*: GGCCATGC**A**GACGCCGACGAAATCTCACTCC （+1 bp） |  |
|  | *oscrk29-36* | *crk29*: GGCCATGC---------CCGACGAAATCTCACTCC （-4 bp） |  |
| ***oscrk29/30*** |  | *OsCRK29*-gRNA1 | *OsCRK29*-gRNA2 |
|  |  | ZH11: GGCCATGCGACGCCGACGAAATCTCACTCC | ZH11: CGCCGGACGTGGTGTACGCCATCGCGCTCT |
|  | *oscrk29/30-57* | *crk29*: WT | *crk29*: CGCCGGAC**T**GTGGTGTACGCCATCGCGCTCT （+1 bp） |
|  | *oscrk29/30-62* | *crk29*: WT | *crk29*: CGCCGGAC**A**GTGGTGTACGCCATCGCGCTCT （+1 bp） |
|  |  | *OsCRK30*-gRNA1 | *OsCRK30*-gRNA2 |
|  |  | ZH11: GGCCATGCGACGCGGGCAACACCTTCCTCT | ZH11: CGCCGGACGTGGTGTACGCCATCGCGCTCT |
|  | *oscrk29/30-57* | *crk30*: WT | *crk30*: CGCCGGAC**A**GTGGTGTACGCCATCGCGCTCT （+1 bp） |
|  | *oscrk29/30-62* | *crk30*: WT | *crk30*: CGCCGGAC**T**GTGGTGTACGCCATCGCGCTCT （+1 bp） |
| ***oscrk31*** |  | *OsCRK31*-gRNA1 | *OsCRK31*-gRNA2 |
|  |  | ZH11: TGCGCCGCCACCGTCCCTACCTCGACGGCG | ZH11: TCCTCGTCTCCGGCCATGTACGCCACGGGC |
|  | *oscrk31-6* | *crk31*: TGCGCCGCCACCGTCCCTA------CGACGGCG （-3 bp） | *crk31*: TCCTCGTCTCCGGCCATGTAC--CCACGGGC （-1 bp） |
|  | *oscrk31-16* | *crk31*: TGCGCCGCCACCGTCCCTACC--CGACGGCG （-1 bp） | *crk31*: TCCTCGTCTCCGGCCATGTAC--CCACGGGC （-1 bp） |
| ***oscrk32/33*** |  | *OsCRK32*-gRNA1 | *OsCRK32*-gRNA2 |
|  |  | ZH11: GGCCTCGCCTTGCTGCTGCTCCACGCGCCC | ZH11: GCCCTCGCGCTCTGTCGTGGCGATACCAAC |
|  | *oscrk32/33-7* | *crk32*: GGCCTCGC--TTGCTGCTGCTCCACGCGCCC （-1 bp） | *crk32*: WT |
|  | *oscrk32/33-20* | *crk32*: GGCCTCGC----TGCTGCTGCTCCACGCGCCC （-2 bp） | *crk32*: WT |
|  |  | *OsCRK33*-gRNA1 | *OsCRK33*-gRNA2 |
|  |  | ZH11: CAAGATCATAAATGGAATAGCTAGAGGGTT | ZH11: GCCCTCGAGTTGATCGATCCATCATTGGGA |
|  | *oscrk32/33-7* | *crk33*: CAAGATCATAAATGGAATAGCT------------------------------------------------------------------------GTTGATCGATCCATCATTGGGA （-728 bp） | |
|  | *oscrk32/33-20* | *crk33*: CAAGATCATA-------------------------AGAGGGTT （-12 bp） | *crk33*: GCCCTCGA----------TCGATCCATCATTGGGA （-5 bp） |
| ***oscrk35*** |  | *OsCRK35*-gRNA1 | *OsCRK35*-gRNA2 |
|  |  | ZH11: CGCCGCCGGCCAGCCGTGGCCGAGCTGCGG | ZH11: CGCCGTCTACGGCGTCATGCTCTGCCGCGG |
|  | *oscrk35-11* | *crk35*: CGCCGCCG**T**GCCAGCCGTGGCCGAGCTGCGG （+1 bp） | *crk35*: CGCCGTCT**G**ACGGCGTCATGCTCTGCCGCGG （+1 bp） |
|  | *oscrk35-13* | *crk35*: WT | *crk35*: CGCCGTCT--CGGCGTCATGCTCTGCCGCGG （-1 bp） |
|  |  |  |  |
|  |  |  |  |
| ***oscrk34/37/38*** |  | *OsCRK34*-gRNA1 | *OsCRK34*-gRNA2 |
|  |  | ZH11: GGCCGCCGCGCTCCCGCTCGCCGCCGGCCA | ZH11: CGCCGACGTCGCCGGCTACGACCGCGCCGT |
|  | *oscrk34/37/38-2* | *crk34*: GGCCGCCG----------CCGCTCGCCGCCGGCCA （-5 bp） | *crk34*: CGCCGACG**T**TCGCCGGCTACGACCGCGCCGT （ +1 bp） |
|  | *oscrk34/37/38-3* | *crk34*: WT | *crk34*: CGCCGACG**A**TCGCCGGCTACGACCGCGCCGT （ +1 bp） |
|  |  | *OsCRK37*-gRNA1 | *OsCRK37*-gRNA2 |
|  |  | ZH11: ACAAGCTTGCTCTGACGGCGCCATGCGGCG | ZH11: CGCCGACGTCGCCGGCTACGACCGCGCGGT |
|  | *oscrk34/37/38-2* | *crk37*: WT | *crk37*: CGCCGACG**C**TCGCCGGCTACGACCGCGCGGT （+1 bp） |
|  | *oscrk34/37/38-3* | *crk37*: WT | *crk37*: CGCCGACG**C**TCGCCGGCTACGACCGCGCGGT （+1 bp） |
|  |  | *OsCRK38*-gRNA1 | *OsCRK38*-gRNA2 |
|  |  | ZH11: CTCCTCTTCGCGGCGGTCGCGCTCCCGCTC | ZH11: CACCGACGTCGCCGGCTACGACCGCGCGGT |
|  | *oscrk34/37/38-2* | *crk38*: WT | *crk38*: CACCGACG**T**TCGCCGGCTACGACCGCGCGGT （+1 bp） |
|  | *oscrk34/37/38-3* | *crk38*: ATGCCG-----------------------------------------------------------------------------------------------------------GCTACGACCGCGCGGT （-435 bp） | |
