# Supplementary material for: An expanded cysteine‐rich receptor‐like kinase gene cluster functionally differentiates in drought, cold, heat, and pathogen stress responses in rice
Source: Plant Biotechnol J. 2024 May 19;22(10):2672–4. doi: 10.1111/pbi.14381 (PMC11536441; doi:10.1111/pbi.14381)
Supplement: Supplementary file 3 — Table S2 List of PCR primers for genotyping of oscrk mutants. [file PBI-22-2672-s002.docx]

**Table S2. List of PCR primers for genotyping of *oscrk* mutants.**

| **Gene** | **Forward primer sequence** | **Reverse primer sequence** |
| --- | --- | --- |
| *OsCRK12* | 5’-GTCCAAGCTAGAGCCGAAGTCAC-3’ | 5’-AGTTCCATTGATGCTGGCCGTGT-3’ |
| *OsCRK13* | 5’-TTAGGCTTAGCCATTCCATTCCATG-3’ | 5’-GGAGCTGGTACTGGTCGTAGTAGACG-3’ |
| *OsCRK14* | 5’-AGCTGAGAAAGAAACACGCACTAATCA-3’ | 5’-CGAGTTGTACGACCCGTACATGGC-3’ |
| *OsCRK15* | 5’-CGGGATGAACAGGGAACACCACT-3’ | 5’-ATCGGAATCTAACAGAACATTACTTGC-3’ |
| *OsCRK16* | 5’-TATTCTTTGTCGTCTGACCCG-3’ | 5’-TGCACGTCGTACCAGTAGTT-3’ |
| *OsCRK17* | 5’-ACAGGTGACGAGTGACGACGGGTCT-3’ | 5’-GGTCATGTTTGCTGGTCCAGGTGC-3’ |
| *OsCRK18* | 5’-GCAGTAGGGGTCCTATACGTCAGTC-3’ | 5’-GTCGTTTGGTTGTCTTCATTTTGTC-3’ |
| *OsCRK19* | 5’-GGCATTTCCGTGTCGGAAGGTGA-3’ | 5’-GTGATGCTGTCGGAATCGGTGGC-3’ |
| *OsCRK20* | 5’-CGCAAGGTTCGTCCGTCAAAGAAG-3’ | 5’-GCCCTGATAGAACGGCTTCACGCT-3’ |
| *OsCRK22* | 5’-TCAAATTCACTGCTAAACCGGTATCTT-3’ | 5’-GCCAGGCTGTACACCTTGGGGAG-3’ |
| *OsCRK23* | 5’-TCAAACTCAGCACAACGTGCCTAATG-3’ | 5’-GTCGAACTCCTCCTCACCCGTCA-3’ |
| *OsCRK24* | 5’-ACAGACCACCCGTTCATCCGTTC-3’ | 5’-GTCTGGTTAACTGCGGGCACCAC-3’ |
| *OsCRK25* | 5’-AATCTGCCATTTGACAAGCGATGTG-3’ | 5’-CGGGATACCAACTGTTTGTAACGAGCT-3’ |
| *OsCRK26* | 5’-GGTTGAATGCAACTTCAAGTGGGC-3’ | 5’-GGATATGGTTAGGAAGAAATGGTCGG-3’ |
| *OsCRK27* | 5’-GAGCAAGAGCAAACCACCTTCTTCTT-3’ | 5’-GACGGTCCCGGTGTTGTAGAGGT-3’ |
| *OsCRK29* | 5’-TTTCCTCTTCTTCCGAGTATTTATGGC-3’ | 5’-AGTAGCGGAGGATACAGGGGTCG-3’ |
| *OsCRK30* | 5’-TGCTCCTACAAATAAGTTGTGCGTTT-3’ | 5’-AGGATGTCCAGGTCGGAGTAGCG-3’ |
| *OsCRK31* | 5’-AACAAATGATGTGCCTAAACCACTTTC-3’ | 5’-GGAGCTGGCAGAGGTCGTAGAAGA-3’ |
| *OsCRK32* | 5’-TGGAAGTCCACTGTCCACGCATAC-3’ | 5’-GCAACGACCATCCCTTGGCTATT-3’ |
| *OsCRK33* | 5’-TTTTGGTACTATTTCTCACAGCTCCCT-3’ | 5’-TCGGTGCTTCACTTTGATACATTTT-3’ |
| *OsCRK34* | 5’-CATGGCAACTTGCCAACACCATC-3’ | 5’-GCGGGAACGTCTTCCACCACCTG-3’ |
| *OsCRK35* | 5’-AGCTTACTAGCTTTCCATCATCCCACC-3’ | 5’-GTCGGAGACGCGGACGTAGCACT-3’ |
| *OsCRK37* | 5’-GGACGTCCACTGGCTATTAAAACAGTT-3’ | 5’-AGCTCGGACCTCAGGTAGCACCG-3’ |
| *OsCRK38* | 5’-ATATGCCAGTTGGGATTATGTATTAAT-3’ | 5’-CTGCGCCATGGAGTAGATGTTGC-3’ |
